# Commentary on “Platelet Studies in Autism Spectrum Disorder Patients and First-Degree Relatives”

**DOI:** 10.1186/s13229-016-0086-8

**Published:** 2016-03-29

**Authors:** George M. Anderson, Edwin H. Cook

**Affiliations:** Yale Child Study Center, Yale University School of Medicine, 230 South Frontage Rd., 06519 New Haven, CT USA; Department of Laboratory Medicine, Yale University School of Medicine, New Haven, CT USA; Institute for Juvenile Research, Department of Psychiatry, University of Illinois at Chicago, Chicago, IL USA

**Keywords:** Serotonin, Autism, Platelet aggregation, Platelet release, Hyperserotonemia, Platelet functioning, Platelet count, 5-HT

## Abstract

We comment on the recent report entitled “Platelet Studies in Autism Spectrum Disorder Patients and First-Degree Relatives” [Molecular Autism 2015;6:57]*.* We find it commendable that the authors have investigated platelet factors potentially involved in the well-replicated observation of platelet hyperserotonemia in autism. However, we believe the results need a fuller discussion in the context of prior studies, think that certain aspects of the interpretation need to be reassessed, and attempt to provide a framework for further research in this area.

## Commentary

We would like to comment on the recent paper of Bijl and colleagues entitled “Platelet Studies in Autism Spectrum Disorder Patients and First-Degree Relatives” [1]. We think the authors should be commended for attempting to understand the mechanism(s) underlying the well-replicated platelet hyperserotonemia of autism, for their focus on platelet functioning, and for the size of the sample studied. However, we believe that (1) the comparison to prior studies of platelet counts, morphology, and functioning in pediatric and autism and autism spectrum disorder (ASD) samples should be more complete and (2) some aspects of the interpretation of results need to be corrected. Before discussing specific findings and issues, we will give a brief overview of the origins and disposition of blood, plasma, and platelet serotonin (5-hydroxytryptamine (5-HT)) and of the platelet hyperserotonemia of autism.

### Platelet 5-HT

Nearly all blood 5-HT is located in the platelet, with only about 0.1 % usually being found free in the plasma. Thus, concentrations of 5-HT in carefully prepared and accurately assayed platelet-poor plasma (PPP) appear to be approximately 100–200 pg/mL compared to typical group mean whole blood levels in healthy controls of about 150 ng/mL (~1000-fold higher than PPP). When expressed on a per platelet basis, mean 5-HT levels in whole blood or in platelet-rich plasma are about 600 ng per billion platelets (i.e., typical adult whole blood platelet counts are 0.25–0.30 billion/mL and the PRP platelet count is often adjusted to 0.25 billion/mL before use in platelet function tests). The 5-HT found in blood/platelets originates predominantly from enterochromaffin cells of the gut and is taken up from the plasma throughout the platelets’ 8- to 10-day lifespan via the 5-HT transporter. Platelet 5-HT is localized in the dense granule and is released along with ATP during aggregation and can augment aggregation through the stimulation of platelet membrane 5-HT2A receptors.

### Hyperserotonemia of autism

Group mean concentrations of blood 5-HT in ASD groups are typically reported to be 25–50 % higher than in healthy controls, and levels do not appear to be related to intellectual disability [2–6]. Although this “hyperserotonemia” was first reported in 1961 [7] and has been thoroughly characterized over the years, there is still frustratingly little that can be said about the mechanism of the elevation. Simply put, it can be suggested that the elevation in 5-HT is either due to the platelet being exposed to higher levels of 5-HT or to some alteration in the platelet or its handling of 5-HT. The measures employed by Bijl et al. are relevant to both of these possibilities.

### Observation of increased platelet count in ASD

Bijl and colleagues observed group mean whole blood platelet counts that were 12 % higher in ASD compared to pediatric controls. Some prior studies have also reported slight but statistically significant elevations in mean platelet counts in autism [4, 8], while others have not observed significant differences in platelet count [9, 10]. Some of the discrepancies might be due to the reported substantial effects of age and/or puberty, with lower platelet counts observed for adolescents and young adults compared to pre-pubertal children (e.g., [4, 10, 11]). Given the substantial difference in mean age in their ASD and pediatric control groups (11.9 versus 15.6 years), it would have been desirable for Bijl and colleagues to covary or better match for age and to make separate diagnostic group comparisons within the pre and the post-pubertal subgroups. Their finding of only a slight and non-significant trend to lower mean platelet volume (MPV) in ASD is consistent with most prior reports. When McBride et al. [4] calculated the total platelet volume (TPV) by multiplying an individual’s mean platelet volume by the platelet count; mean TPVs were very similar in ASD and control groups. To summarize, it appears that little if any of the increase in platelet 5-HT in autism can be attributed to either increased platelet counts or differences in platelet size.

### Observation of higher platelet-poor plasma 5-HT in ASD

It should be pointed out that the values for PPP 5-HT (~10–20 ng/mL) reported in Bijl et al. are much too high to represent in any manner the much lower levels of 5-HT actually present in PPP. As has been previously discussed at length [12, 13], difficulties in preparing PPP without any platelet contamination or platelet 5-HT release and problems with accurately measuring the low levels of 5-HT present have made nearly all prior measurements of PPP 5-HT falsely and markedly elevated and useless. The two studies providing the apparent best estimates of PPP 5-HT in autism (and thereby an index of platelet exposure to 5-HT) found similar levels in autism and contrast groups [14, 15]. As stated above, the finding of higher whole blood or platelet 5-HT in ASD has been well replicated, and it is safe to assume that the ASD group studied by Bijl and colleagues also had an elevated group mean platelet 5-HT concentration. However, their reported PPP values say nothing about the extent of the (presumed) 5-HT elevation in this group or about the platelet or PPP 5-HT levels in a particular individual. While their PPP 5-HT measurements and the associated correlations and group comparisons should be disregarded, the authors are almost certainly correct in saying that their observation of comparatively greater release and aggregation in the control group in response to epinephrine, ADP, or collagen cannot be due to increased plasma 5-HT levels in controls.

### Observation of lower aggregation and release in ASD

As just mentioned, Bijl and colleagues observed that the ASD group had lower platelet aggregation and dense granule (ATP) release in response to epinephrine, ADP, and collagen stimulation. As the authors acknowledge, these observations are confounded by the lack of an age-matched (pediatric) control group for the aggregation and release experiments. However and on their behalf, it can be pointed out that there do not appear to be age-related changes in platelet responses to aggregating agents after the neonatal period to the extent this has been studied in platelet-rich plasma [16] and in whole blood [17]. It would have been informative to compare their findings to those of Saffai-Kutti et al. [18] and McBride et al. [19], the latter of whom reported reduced 5-HT augmentation of ADP-induced aggregation in ASD versus controls. This lower augmentation was observed without any group difference in the concentration of ADP required to produce a sub-threshold (15–20 %) aggregation response. Although the McBride et al. observation of similar sensitivity to ADP is at some variance with the Bijl et al. report of lower ADP-induced aggregation and release, it would be of fundamental interest to assess the relationship between platelet levels of 5-HT and the aggregation and release responses. In the McBride et al. report, an inverse relationship was seen between platelet 5-HT level and blunted response to 5-HT augmentation, leading to the suggestion that reduced platelet 5-HT2A receptor functioning might somehow lead to greater platelet 5-HT [19]. As an aside, it would also be of interest to know what sort of parent-sib and sib-sib correlations are found with the platelet functioning measures given the high heritability reported for platelet 5-HT [20].

### Interpretation of platelet functioning results

We find it necessary to correct the statement of the discussion that reads “Since we find reduced platelet aggregation and secretion in ASD, we hypothesize that the increased serotonin levels are caused by reduced uptake rather than by increased release from platelets.” The authors appear to have confused platelet and plasma 5-HT and have failed to appreciate that while platelet 5-HT is consistently found to be elevated in ASD groups, the miniscule PPP 5-HT values are probably not increased in ASD [14, 15]. It is clear that the increased platelet 5-HT levels of ASD can in no way be attributed to either reduced uptake or increased release. Rather, and quite the contrary, it is possible that either increased uptake or decreased release, or both, could contribute to the platelet hyperserotonemia. While studies of platelet 5-HT uptake rates and 5-HT transporter sites, as well as associated genes, have suggested that increased uptake might contribute to the hyperserotonemia, the extent of that contribution remains unclear (for reviews, see references [2, 3, 21, 22]).

Although we find some problems with the Bijl et al. paper, we do think the simultaneous measurement of 5-HT levels and of indices of platelet functioning is a valuable approach. The paper has served to bring attention to important issues related to possible mechanisms of the platelet hyperserotonemia of ASD and to the potential utility of the platelet measures in ASD. It is clear that, along with the platelet functioning measures, future studies in this area should assay platelet 5-HT levels and that this is probably best and most simply done using whole blood samples. Concomitant careful measurements of PPP 5-HT would add to the only limited data presently available regarding the platelet’s exposure to 5-HT. A possible contributor to the platelet hyperserotonemia of autism that has been given little attention is platelet lifespan and its determination would be interest. The search for specific behavioral or biological associations with platelet 5-HT levels in autism has been largely futile to date, but might benefit from new approaches in behavioral assessment and from the application of neurophysiological methodologies.

## Abbreviations

5-HT: 5-hydroxytryptamine
5-HT2A: serotonin type 2A receptor
ASD: autism spectrum disorder
ATP: adenosine triphosphate
MPV: mean platelet volume
ng/mL: nanograms (10^−9^ grams) per milliliter
pg/mL: picograms (10^−12^ grams) per milliliter
PPP: platelet-poor plasma
PRP: platelet-rich plasma
TPV: total platelet volume
